# Diet-Induced Obesity in Male C57BL/6 Mice Decreases Fertility as a Consequence of Disrupted Blood-Testis Barrier

**DOI:** 10.1371/journal.pone.0120775

**Published:** 2015-04-17

**Authors:** Yong Fan, Yue Liu, Ke Xue, Guobao Gu, Weimin Fan, Yali Xu, Zhide Ding

**Affiliations:** 1 Department of Human Anatomy, Histology and Embryology, School of Medicine, Shanghai Jiao Tong University, Shanghai Key Laboratory for Reproductive Medicine, Shanghai, China; 2 Department of Medical Laboratory Science, The Central Hospital of Zhabei District, Shanghai, China; 3 Department of Human Reproductive Medicine, Ruijin Hospital, School of Medicine, Shanghai Jiao Tong University, Shanghai, China; Clermont-Ferrand Univ., FRANCE

## Abstract

Obesity is a complex metabolic disease that is a serious detriment to both children and adult health, which induces a variety of diseases, such as cardiovascular disease, type II diabetes, hypertension and cancer. Although adverse effects of obesity on female reproduction or oocyte development have been well recognized, its harmfulness to male fertility is still unclear because of reported conflicting results. The aim of this study was to determine whether diet-induced obesity impairs male fertility and furthermore to uncover its underlying mechanisms. Thus, male C57BL/6 mice fed a high-fat diet (HFD) for 10 weeks served as a model of diet-induced obesity. The results clearly show that the percentage of sperm motility and progressive motility significantly decreased, whereas the proportion of teratozoospermia dramatically increased in HFD mice compared to those in normal diet fed controls. Besides, the sperm acrosome reaction fell accompanied by a decline in testosterone level and an increase in estradiol level in the HFD group. This alteration of sperm function parameters strongly indicated that the fertility of HFD mice was indeed impaired, which was also validated by a low pregnancy rate in their mated normal female. Moreover, testicular morphological analyses revealed that seminiferous epithelia were severely atrophic, and cell adhesions between spermatogenic cells and Sertoli cells were loosely arranged in HFD mice. Meanwhile, the integrity of the blood-testis barrier was severely interrupted consistent with declines in the tight junction related proteins, occludin, ZO-1 and androgen receptor, but instead endocytic vesicle-associated protein, clathrin rose. Taken together, obesity can impair male fertility through declines in the sperm function parameters, sex hormone level, whereas during spermatogenesis damage to the blood-testis barrier (BTB) integrity may be one of the crucial underlying factors accounting for this change.

## Introduction

Obesity is often defined simply as a status of excessive or abnormal fat accumulation arising from an imbalance between caloric intake and metabolic expenditure [1]. Currently, epidemiological studies show that the proportion of adults with a body-mass index (BMI) of 25 kg/m^2^ or greater significantly increased between 1980 and 2013 worldwide and over 31% of the male adult population in USA is obese in 2013 (defined as a BMI≥30 kg/m^2^) [2]. Moreover, according to the WHO, statisticians have predicted that approximately 2.3 billion adults will be classed as overweight and 700 million adults will be obese in 2015 [3]. It is certain that with the increasing prevalence of unhealthy dietary behaviors and sedentary life styles, obesity is emerging as an important risk factor for non-insulin-dependent diabetes, hypertension, cardiovascular disease, cancer, and relevant metabolic and reproductive disorders [4].

In the past decades, the adverse effects of obesity on female fertility have been well recognized. They include menstrual disorder, anovulation, polycystic ovarian syndrome, an increased risk of miscarriage and a reduced conception rate [5, 6]. Meanwhile, there is increasing evidence that obesity may also impair male fertility [7, 8, 9], although several reports failed to document this association [10, 11]. Notably, clinical data from large-scale epidemiological studies suggested a significant negative correlation between BMI and the semen parameters for evaluation of male fertility potential, including sperm concentration [12], semen volume [13], motility [14, 15] and sperm morphology [16]. Most of studies revealed that spermatogenesis is affected by altered levels of sex hormones in obese men, such as decreased free or total testosterone and increased estradiol levels in serum [13, 17]. Besides, diet-induced obesity is highly susceptible to increases in the DNA fragmentation index in spermatozoa due to oxidative stress, resulting in an obvious decline in male fertility [18]. However, the mechanism is poorly characterized describing how obesity can cause male subfertility and warrants elucidation.

In this study, we initially established a high fat diet (HFD) induced obese animal model in order to determine whether obesity affects declines in male fertility as well as serum reproductive hormone levels and disrupts testicular morphology. Furthermore, during spermatogenesis in obese mice testicular changes in relevant biomarkers of blood-testis barrier (BTB) function were quantified.

## Materials and Methods

### Obese Mouse Model Establishment

All experiments were conducted following the Guide for the Care and Use of Laboratory Animals of National Institutes of Health. This study was approved by the animal ethics committee of Shanghai Jiao Tong University School of Medicine.

Male (3 weeks old) and female (8 weeks old) C57BL/6 mice were purchased from the Shanghai Laboratory Animal Center, and acclimated in the Animal Center of Shanghai Jiao Tong University School of Medicine for one week prior to the research. Male mice were divided in two groups ad libitum: control diet (CD) group containing 19% casein, 0.2% L-cystine, 29.9% corn starch, 3.3% maltodextrin, 33.2% sucrose, 4.7% cellulose, 2.4% soybean oil, 1.9% lard, 4.3% mineral mix, 0.9% vitamin mix, 0.2% choline bitartrate, whereas high-fat diet (HFD) group providing 23.3% casein, 0.3% L-cystine, 8.5% corn starch, 11.7% maltodextrin, 20.1% sucrose, 5.8% cellulose, 2.9% soybean oil, 20.7% lard, 5.2% mineral mix, 1.2% vitamin mix, 0.3% choline bitartrate (Mediscience Ltd, China) in Wu’s formula [19]. All mice were adapted to a 10 hour darkness and 14 hour light cycle at 24°C for 10 weeks and weighed every week.

### Serum Lipid and Sex Hormone Measurements

The mouse serum lipids, including triglycerides (TG), total cholesterol (TC), high density lipoprotein (HDL) and low density lipoprotein (LDL), as well as serum sex hormone levels, such as testosterone (T), estradiol (E2), luteinizing hormone (LH) and follicle-stimulating hormone (FSH) were measured at 14 weeks. Exact levels of lipid in serum were measured using a Roche cobas c 311 autobiochemistry analyzer (Roche Diagnostics, Mannheim, Germany), while levels of testosterone (R&D Systems, USA), estradiol (Cayman chemical, USA), FSH (Elabscience, China) and LH (Shanghai Xinle Biotechnology Ltd., China) in serum were detected using the immunoassay kits according the manufacturer’s protocol, respectively.

### Assessment of Sperm function parameters

The epididymides were separated from mice testes and cauda epididymides were dissected and then placed in pre-warmed (37°C) Tyrode’s Buffer (Sigma-Aldrich, USA) to allow dispersion of sperm. After 15 minutes, sperm motility, progressive motility and concentration were analyzed by computer-assisted sperm analysis (CASA) (Hamilton Thorne, USA).

For teratozoospermia analysis, sperm pellet was initially smeared on a glass slide. After dryness, the slide was fixed and stained by method of Diff-Quick (BRED Life Science Technology Inc., China) according to the manufacturer’s protocol, and finally, the slide was viewed under a microscope (Nikon, ECLIPSE E600, Japan). Sperm samples obtained from three HFD-fed mice and three normal diet-fed mice respectively were detected and at least 200 spermatozoa in every sample were included.

To trigger an acrosome reaction (AR), a sperm suspension was incubated containing 10 μM calcium ionophore A23187 (Sigma-Aldrich, USA) for 1 hour at 37°C in 5% CO_2_. Then a spermatozoa acrosome reaction was evaluated using both Coomassie Blue G250 and FITC-PNA (Sigma-Aldrich, USA) stain respectively. Finally, the Coomassie Blue stained sections and fluorescent stained sections were viewed under a light microscope (Nikon, ECLIPSE E600, Japan) and a laser scanning confocal microscope (LSCM, Carl Zeiss LSM-510, Jena, Germany) respectively. The percentage of acrosome-reacted (AR) cells was calculated on every slide in at least 200 spermatozoa This AR assessment was repeated at least three times from three HFD-fed and three normal diet-fed mice respectively.

### Histological Analysis

After mice were anaesthetized, testes, and livers were immediately excised and weighed. One testis was stored at -80°C until later for protein extraction. The other testis and a small piece of liver tissue were fixed in Bouin’s Solution for 12h, and then stored in 70% ethanol for 2 hours. Tissues were embedded in paraffin, and sliced into 7 μm thickness sections, and mounted on polylysine sides followed by dewaxing and rehydration. The slices were then stained with hematoxylin and eosin (H&E). Images were captured using a microscope (Nikon, ECLIPSE E600, Japan).

Furthermore, testicular tissues were examined using a transmission electron microscope. The small pieces of testicular tissue were immersed in 2% glutaraldehyde solubilized in 0.1 mol/L phosphate buffer (pH 7.4) for 1 day, and post-fixed in 1% osmium tetroxide. Dehydration was carried out in a graded ethanol series, and then samples were embedded in Epon 618 (TAAB Laboratories Equipment, Berks, UK). Ultra-thin sections (70–90 nm) of the seminiferous epithelium region were stained with lead citrate and uranyl acetate, and then checked under a transmission electron microscope (PHILIPS CM-120, Eindhoven, Netherlands) at 80 kV.

Tissues obtained from three HFD-fed and three normal diet-fed mice respectively were examined and at least ten visual fields were viewed for every sample to confirm the results of morphological analysis.

### BTB Integrity Assay

The integrity of the BTB was assessed as previously described [20, 21]. Briefly, male mice were anesthetized, and then 200 μl of 5 mg/ml fluorescein isothiocyanate isomer I (Sigma-Aldrich, USA), freshly diluted in PBS, were injected into the caudal vein of mice. After two hours, the mice were euthanized and their testes were immediately removed and embedded in OCT (Sakura). Cryosections with 8μm thickness were obtained in a cryostat microtome and fluorescence images were acquired by a laser scanning confocal microscope (LSCM, Carl Zeiss LSM-510, Jena, Germany).

### Mating Assay

At fourteen weeks of age, mice that had been individually housed were cohabited for 5 consecutive days with two female C57BL/6 mice. Vaginal plugs were counted every morning after pairing to determine if mating had occurred. Each mated mouse was isolated to a separate cage. Approximately twenty-one days after the last day of cohabitation, the number of pups delivered by each mated female mouse was counted and the litter sizes of both HFD and control groups were analyzed.

### Preparation of Protein Specimens and Western Blotting

Testes placed in lysis buffer containing RIPA buffer and protease inhibitor cocktails were dispersed with homogenizers (Thermo Fisher Scientific, USA) on ice. Then the lysates were centrifuged at 12,000 × g, 10 min, 4°C, and the supernatant containing testicular proteins were stored immediately at -80°C until later use. Protein concentrations were determined by using the BCA^TM^ Protein Assay Kit (Thermo Fisher Scientific, USA).

SDS-PAGE was conducted on 30 μg testicular protein per well using 10% denaturing polyacrylamide gels and then proteins were electrotransferred to PVDF membranes, using a semi-dry transfer apparatus [22]. Membranes were blocked for 1 h at room temperature with 5% bovine serum albumin (BSA) and immunoblotting was performed overnight at 4°C with one of the following antibodies: occludin (Invitrogen, 1:1000), ZO-1 (Invitrogen, 1:1000), androgen receptor (Santa Cruz, 1:500), clatherin (Invitrogen, 1:1000), followed by incubation with secondary antibody conjugated to HRP at a 1:8000 dilution. After washing with Tris-buffered saline (TBS), signals were detected by enhanced chemiluminescence according to the manufacturers’ instructions. Meanwhile, β-actin served as the internal control. Western blot was repeated at least three times for each sample from three HFD and CD mice, respectively.

### Statistical Analysis

All data were analyzed using SAS 8.2 software, and results are expressed as mean ± SEM. Comparisons between two groups were made using Student’s t-test, as appropriate. One-way analysis of variance (ANOVA) test was used assuming a two-tail hypothesis with *P*<0.05.

## Results

### Body Weight of Experimental Animals

**Fig 1:**
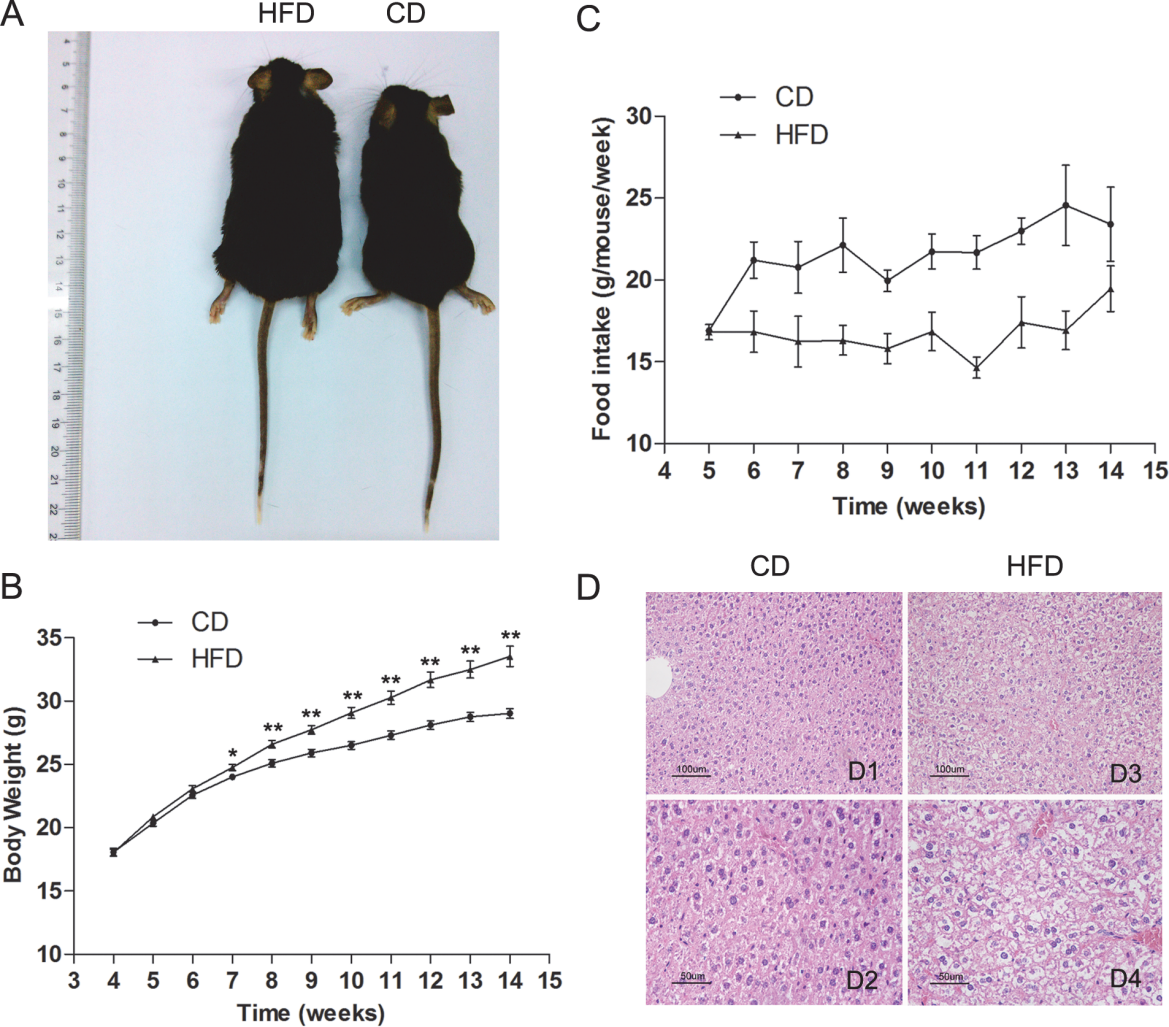
Description of an obese mouse model established after 10 weeks on HFD. (A) Representative picture of 15-week old obese and control mice. (B) Comparison of time-dependent increases in body weight between CD (n = 27) and HFD groups (n = 30). (C) Weekly food consumption by mice in CD and HFD groups. (D) Hematoxylin and eosin-stained hepatic sections from mice both fed control diet (CD) and high-fat diet (HFD). (D2) and (D4) are two-fold enlarged views of (D1) and (D3). Scale bars = 50 μm. Data are expressed as mean ± SEM. * *P*<0.05, ** *P*<0.01.

Male C57BL/6 mice fed a high-fat diet for 10 weeks gained significantly more body weight than their age-matched littermates fed a normal diet (33.54 ± 0.81 vs. 29.02 ± 0.38, n = 30, *P*<0.01). The difference in body weight between these two groups became significant after 3 weeks being fed their designated diets and persisted for the subsequent seven weeks as shown in Fig 1A and 1B. The feed intake per week (g/week) of the HFD group was always less than that of the CD group (Fig 1C). However, the high-fat diet had a much higher caloric content than the normal diet.

### Effect of HFD on Serum Lipids and Sex Hormones

**Fig 2:**
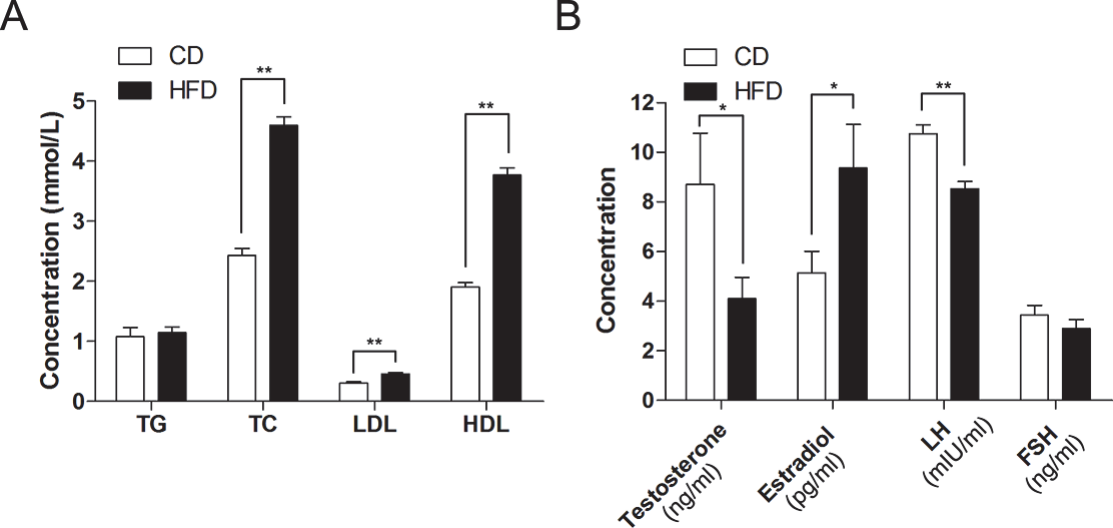
Alteration of serum lipids and sex hormone levels in HFD mice. (A) Comparison of serum lipid levels between CD and HFD groups; TG: triglycerides, TC: total cholesterol, LDL: low density lipoprotein, HDL: high density lipoprotein. (B) Comparison of serum sex hormone levels between CD and HFD groups, including testosterone, estradiol, luteinizing hormone (LH) and follicle-stimulating hormone (FSH). Data are expressed as mean ± SEM. * *P*<0.05, ** *P*<0.01.

Male mice fed the HFD had significantly higher levels of total cholesterol (TC, 4.60 ± 0.14 vs. 2.43 ± 0.11, n = 10, *P*<0.01), low-density lipoprotein (LDL, 0.46 ± 0.02 vs. 0.30 ± 0.02, n = 10, *P*<0.01) and high-density lipoprotein (HDL, 3.77 ± 0.12 vs. 1.90 ± 0.07, n = 10, *P*<0.01) than those in the control group, but there was no difference between the triglycerides levels given to the two groups (TG, 1.14 ± 0.1 vs. 1.08 ± 0.15, n = 10, *P*>0.05) (Fig 2A). Besides, morphological analysis of liver sections clearly indicated that the HFD fed mice had a serious hepatic steatosis and fat vacuoles were evident in almost every hepatic cell (Fig 1D).

Serum estradiol levels were significantly elevated in obese mice compared to those in the control group (9.36 ± 1.76 vs. 5.14 ± 0.87, n = 26, *P*<0.05), while serum testosterone (4.11 ± 0.86 vs. 8.71 ± 2.07, n = 18, *P*<0.05) and luteinizing hormone (LH, 8.53 ± 0.30 vs. 10.80 ± 0.36, n = 18, *P*<0.01) levels were lower in obese mice than those in the control group. However, follicle-stimulating hormone (FSH) levels were unaltered in the two groups (2.90 ± 0.36 vs. 3.43 ± 0.41, n = 10, *P*>0.05) (Fig 2B).

### Alteration of Sperm Function Parameters and Morphology in Obese Mice

**Fig 3:**
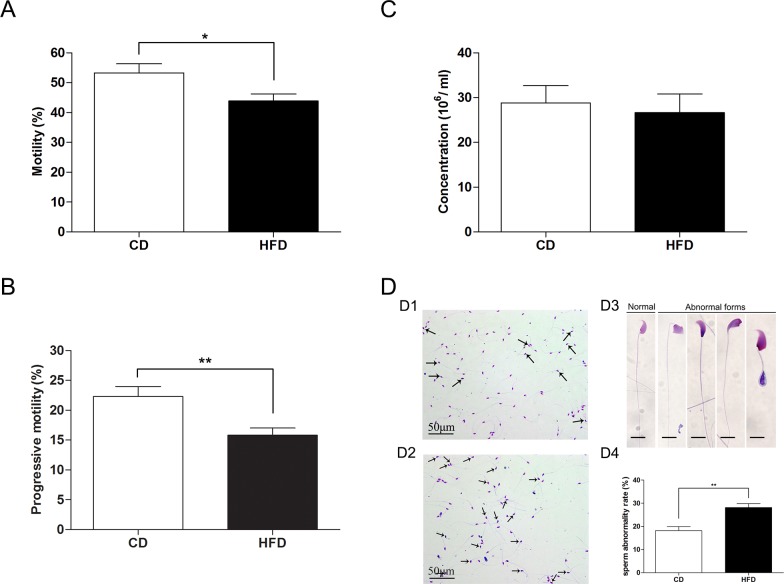
Comparison of sperm function parameters in mice fed either CD or HFD. (A) Sperm motility in mice fed either CD or HFD analyzed by CASA. (B) Sperm progressive motility in mice fed either CD or HFD analyzed by CASA. (C) Sperm concentration from cauda epididymis in mice fed either CD or HFD analyzed by CASA. (D) Sperm morphology analysis of CD-fed mice (D1) and HFD-fed mice (D2) using Diff-Quick method. Arrows indicate abnormal spermatozoa. Scale bar = 50 μm. (D3) Normal and abnormal spermatozoa in different forms, Scale bar = 10 μm. (D4) Comparison of sperm abnormality in mice fed either HFD or CD. Data are expressed as mean ± SEM. *P<0.05, **P<0.01.

CASA analysis of spermatozoa extracted from the cauda epididymides revealed that the percentage of motility and progressive motility significantly decreased in the HFD group when compared to those in the CD group (percentage of motility: 43.85 ± 2.35 vs. 53.27 ± 3.06; progressive motility: 15.82 ± 1.19 vs. 22.32 ± 1.67, n = 27, *P*<0.05) (Fig 3A and 3B). However, sperm concentration of HFD group was not significantly altered in comparison to that in the CD group (26.65 ± 4.18 vs. 28.83 ± 3.84, n = 27, *P*>0.05) (Fig 3C). Moreover, we also observed that there was a higher ratio of teratozoospermia in the HFD group compared to that in the CD group (Fig 3D1–3D4; 28.17 ± 1.64 vs. 18.17 ± 1.74, n = 3, *P*<0.05).

**Fig 4:**
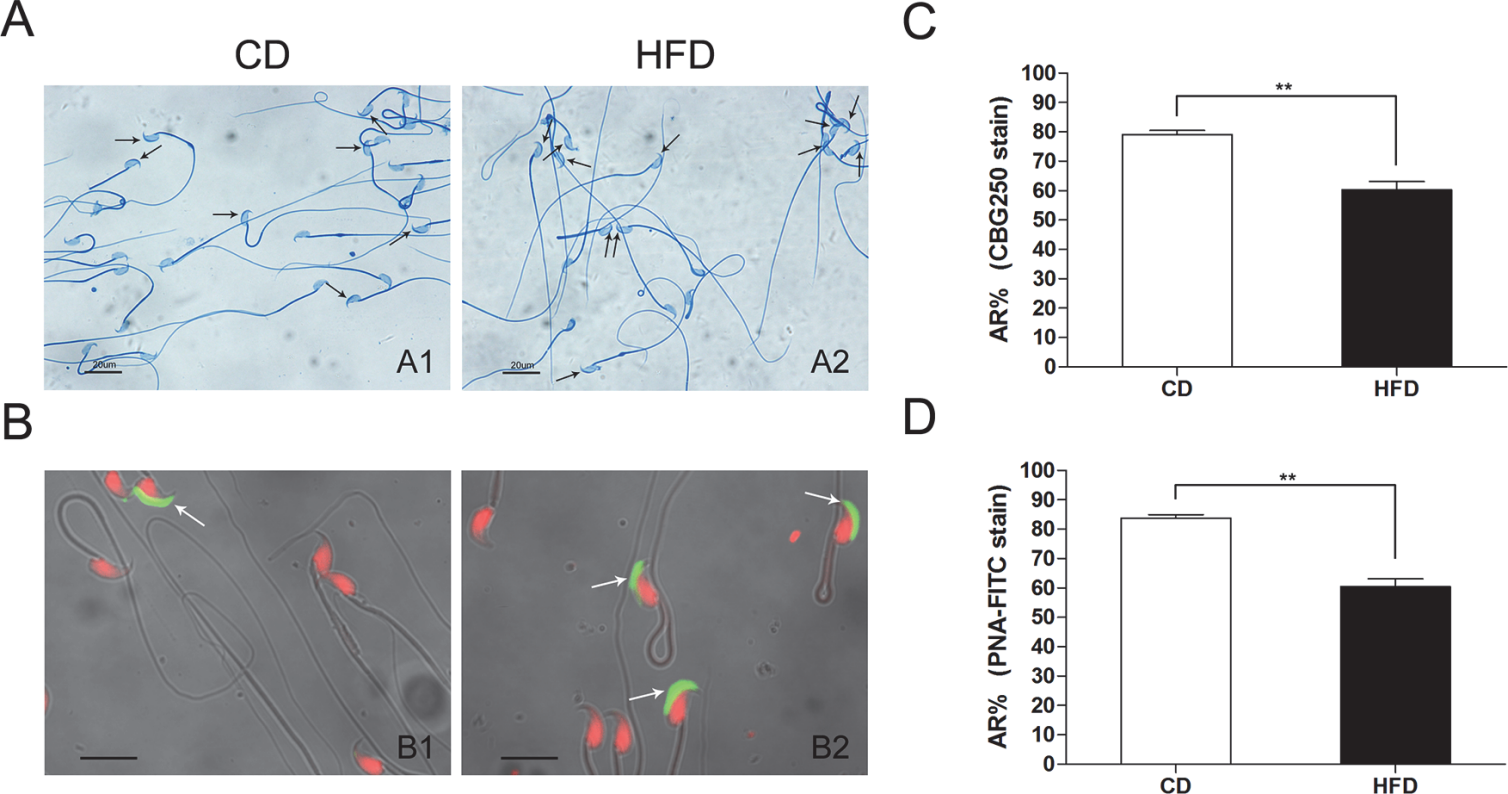
Effects of HFD on sperm acrosome reaction in mice. (A) Status of the mouse sperm acrosome stained with Commassie blue G250 in HFD and CD groups; the arrow indicates the intact acrosome stained with intensive blue. Scale bar = 20 μm. (B) Status of the mouse sperm acrosome stained with FITC-PNA, the arrow indicates the intact acrosome exhibiting green fluorescence. The red fluorescence indicates sperm nuclei stained with Propidium Iodide. Scale bar = 10 μm. A1 and B1: spermatozoa from CD mice; A2 and B2: spermatozoa from HFD mice. AR: acrosome reaction; CBG250: Commassie blue G250. Data are expressed as mean ± SEM. **P<0.01.

To examine whether the effect of HFD on the sperm AR, we evaluated the sperm acrosome status induced by A23187 using two routine staining methods. With Commassie blue G250 stain, the acrosome regions of acrosome-intact spermatozoa were stained intensively blue, whereas acrosome-reacted spermatozoa lacked blue staining in the acrosome area (Fig 4A). Meanwhile, in FITC-PNA stain, the acrosomes of acrosome-intact spermatozoa exhibited a green fluorescence (Fig 4B). Acrosomes stained by FITC-PNA had a lower percentage of AR in the HFD group than that in the CD group (60.31 ± 2.84 vs. 79.13 ± 1.44, n = 6, *P*<0.01) (Fig 4D), which is consistent with the results obtained from spermatozoa stained with Commassie blue G250 (60.42 ± 2.70 vs. 83.8 ± 1.12, n = 6, *P*<0.01) (Fig 4C).

### Effects of HFD on Sperm Fertility in Vivo

To further determine the effect of a HFD on male fertility in a physiological context, we compared the fertility rates of male CD and HFD mice through natural mating. Only 43.0% ± 3.5% (n = 46) of female mice mated with HFD males produced offspring, whereas 71.1% ± 4.4% (n = 46) mated with the age matched CD males. This fact indicates the significant decrease in fertility of HFD mice. However, there was no difference in the number of pups per litter between females mated with CD and those mated with HFD mice (6.34 ± 0.94 vs. 6.76 ± 0.35, n = 46, *P*>0.05) (Table 1).

**Table 1:** Comparison of fertility in CD and HFD mice.

	Male mice	Female mice	Fertility rate (%)	Pups per litter
CD	n = 23	n = 46	71.1± 4.4 (32)	6.34 ± 0.94
HFD	n = 23	n = 46	43.0 ± 3.5 (20) **	6.76 ± 0.35

**P < 0.01

### Alteration of Testicular Morphology and BTB Integrity in Obese Mice

**Fig 5:**
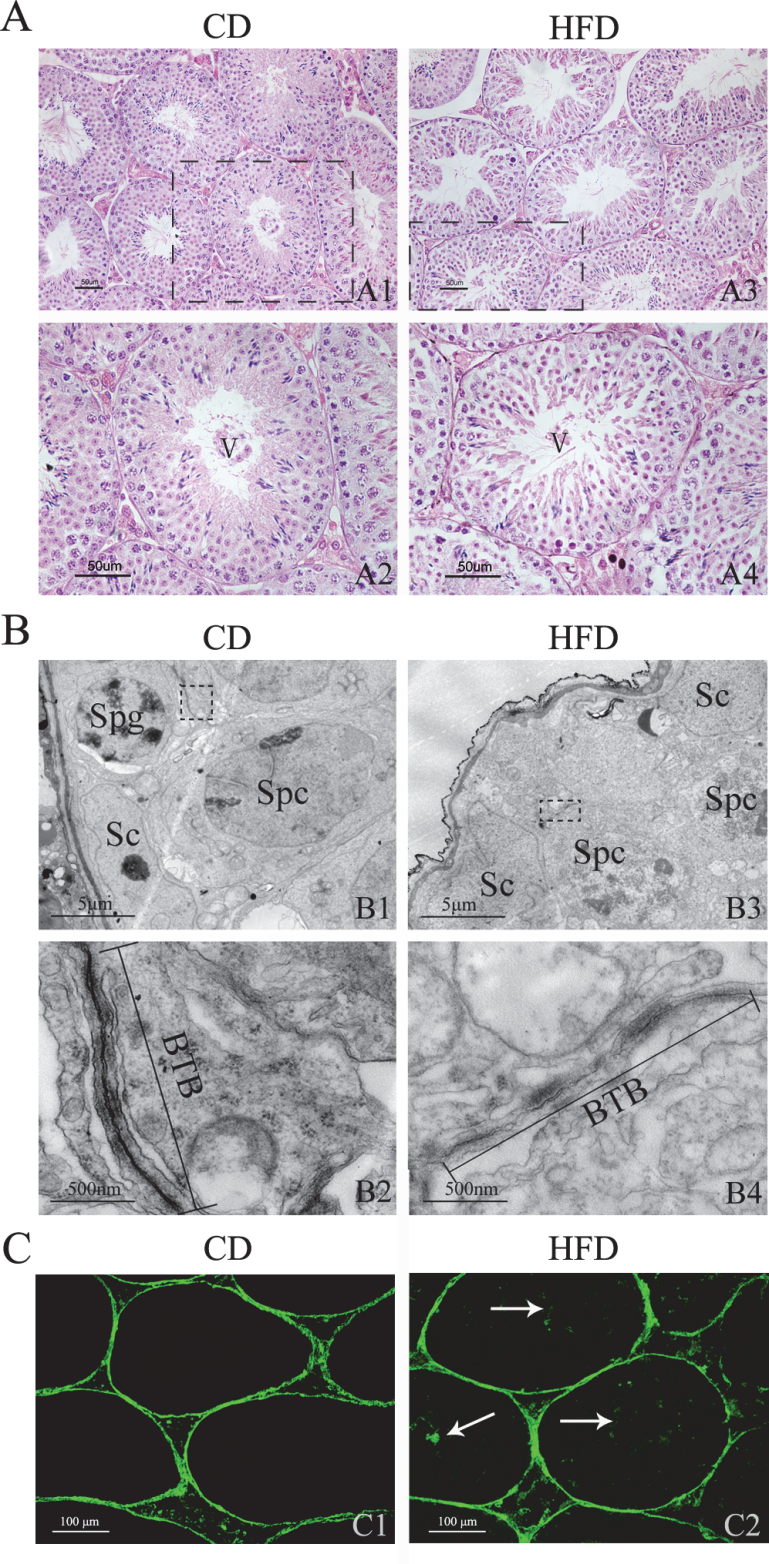
Effects of HFD on mouse testicular morphology and BTB integrity. Hematoxylin and eosin-stained testicular sections from mice fed CD (A1) and HFD (A3). Scale bars = 50 μm. (A2), (A4) Enlarged views of indicated sections in (A1) and (A3); the sections of seminiferous tubules are both in spermatogenic stage V. Scale bars = 50 μm. (B) Transmission electron micrograph of seminiferous epithelium of mice fed CD (B1) and HFD (B3). Sc, Sertoli cell; Spg, spermatogonium; Spc, primary spermatocyte. Scale bars **=** 5 μm. (B2), (B4) Magnified views of indicated sections in (B1) and (B3). Straight lines show BTB between two adjacent Sertoli cells constituted by cellular tight junctions. Scale bars = 500 nm. (C1) In the sections of testes from CD mice, the FITC green fluorescence is only observed in the interstitial spaces and basal compartment. (C2) In the sections of testes from HFD mice, besides the interstitial space and basal compartment, the FITC green fluorescence is also viewed in the lumen of seminiferous tubules (arrows). Scale bars = 100 μm.

Testicular morphological analysis showed that seminiferous epithelia in HFD mice were severely disorganized and atrophic, and cell adhesion between Sertoli cells and spermatogenic cells was disrupted and loosely arranged (Fig 5A, n = 3). To characterize the abnormalities in HFD mice spermatogenesis at the ultrastructural level, TEM analyses of testis from both CD and HFD mice were performed. As depicted in Fig 5B, Sertoli cells in CD mice were well organized, and the BTB was lined with endoplasmic reticulum cisternae and clearly delimited (Fig 5B1 and 5B2). In contrast, the cell junctions adjoining Sertoli cells appeared discontinuous in the seminiferous tubules from HFD mice, indicating that the integrity of BTB was severely compromised (Fig 5B3 and 5B4, n = 3). On the other hand, FITC green fluorescence was detected in the interstitial space and basal compartment in the testes of CD mice (Fig 5C1), whereas the green fluorescence was clearly viewed in the lumen or adluminal compartment of most seminiferous tubules in the testes of HFD mice (Fig 5C2), which indicated that the integrity of the BTB in the testes of HFD mice was indeed disrupted.

### Changes in Androgen Receptor, Clathrin and Tight Junction-associated Proteins after HFD exposure

**Fig 6:**
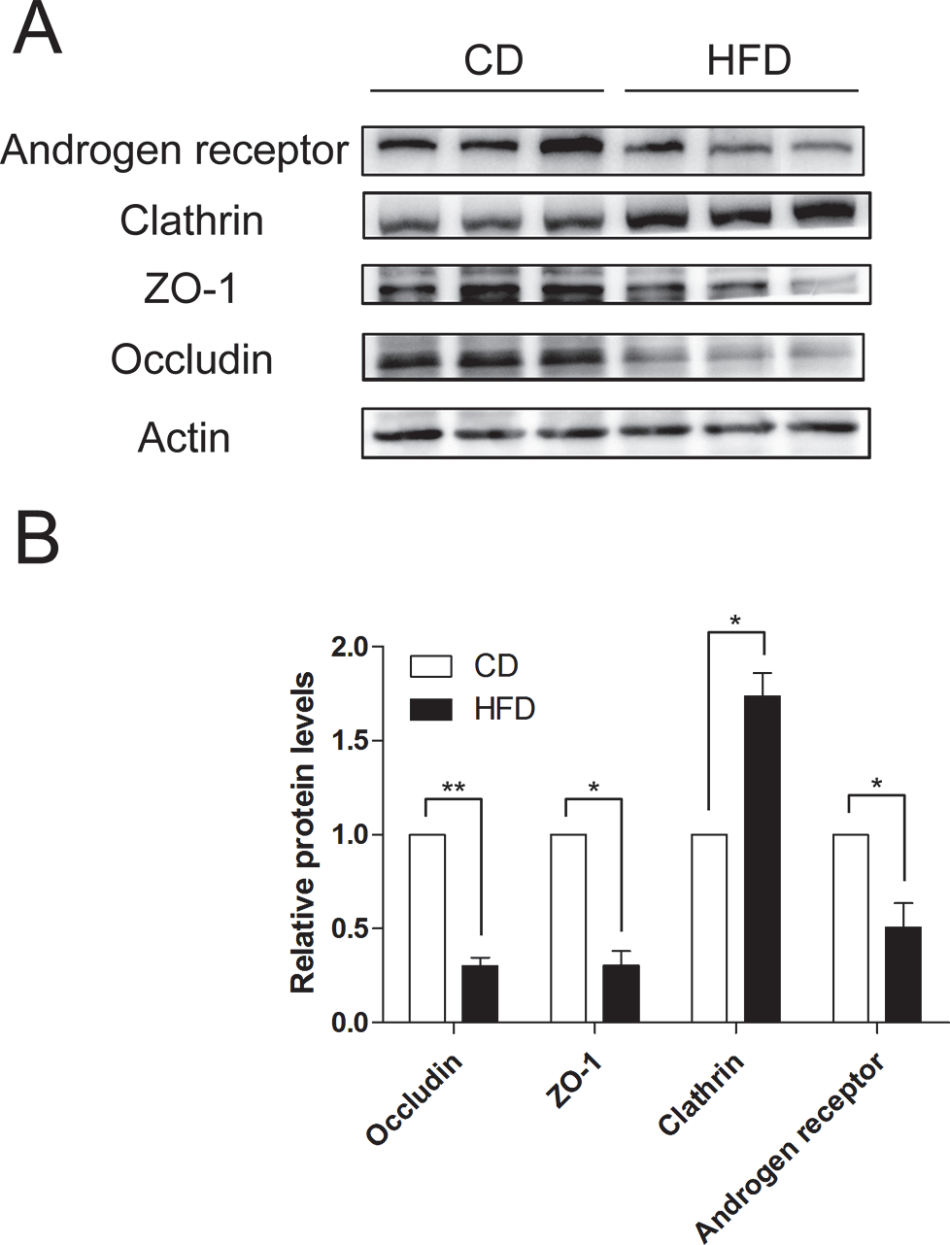
HFD-induced androgen receptor, clathrin, ZO-1 and occludin expression changes in mice. (A) Western blot analyses of androgen receptor, clathrin, ZO-1 and occludin expression levels in testicular protein from CD and HFD mice. (B) Summary plot showing densitometric readings of the corresponding protein blots. Data are expressed as mean ± SEM. *P<0.05, **P<0.01.

To validate disruption of BTB integrity after HFD exposure, we investigated tight junction-associated protein levels in the testes of CD and HFD mice. The results demonstrate that levels of tight junction-related biomarker proteins, such as occludin and zonula occludens 1 (ZO-1), decreased in mice on a HFD. Besides, one of the other consequences of being on a HFD besides BTB disruption was a decrease in androgen receptor expression. On the other hand, another crucial related factor of testicular integrity, the expression of an endocytic vesicle-associated protein, clathrin, increased significantly in the HFD group (Fig 6, n = 3).

## Discussion

The incidence of obesity is increasing world-wide at near epidemic proportions as a critical cause of several adverse health outcomes, including male infertility [23]. In humans, many previous studies focused on the effects of BMI on the semen parameters or serum sex hormones [8, 13], but the mechanism by which male fertility is severely impaired, especially relevant to spermatogenesis or spermatozoa development is still unclear.

To probe for the pathogenic factors that lead to male reproductive dysfunction, we initially established an obese mouse model induced by a high-fat diet. This was done in the hope of mimicking the pathophysiology induced by obesity in humans [24]. Plenty of lard in HFD may be a critical factor to cause metabolic diseases, which is attributed to its high saturated fatty acids content, and our results also indicate that the high-fat diet that we used promoted obesity development since rises in serum lipid levels along with total cholesterol, LDL and HDL accompanied significant weight gain. These changes were considerably aggravated by a long-term ingestion of the HFD, and pathological analysis revealed fatty liver development was accompanied by serious hepatic steatosis.

On the other hand, endocrine dysregulation is another typical characteristic of obesity. Clinical analysis of obese showed that fatty tissue accumulation was directly associated with declines in serum levels of total and free testosterone (T) and increases in estradiol (E2) levels [25, 26, 27], which concurs with our results. These inverse changes in testosterone and estradiol levels may be a consequence of rises in aromatase levels expressed at high levels in white adipose tissue. This enzyme is responsible for a key step in the conversion of androgens into estrogens and it can directly lead to lower serum testosterone levels along with higher estradiol levels in the HFD mice [28, 29]. Besides, estrogen has a negative effect in the hypothalamus through gonadotropin-releasing hormone (GnRH) pulse regulation. Rises in estrogen elicit declines in GnRH release leading to suppression of both LH and FSH secretion, which in turn reduce testosterone and spermatozoa production [23, 30]. Although serum FSH level decreased in the HFD group, this decline did not reach statistical significance. It is very likely that FSH levels may actually significantly fall during a longer period of exposure to the HFD.

To confirm the relationship between obesity and male fertility, we examined semen parameters affected by obesity. CASA analysis showed that mice fed a HFD have impaired spermatozoa as indicated by a significant reduction in motility and progressive motility. However, there is no difference in sperm concentration between HFD and control mice, which is consistent with previous studies in the human [31] and mouse [32, 33]. On the other hand, the HFD mice have a significantly higher percentage of teratozoospermia compared with that in CD mice, which suggests that obesity is definitely a detriment to spermatogenesis. Besides, as a precondition for fertilization, sperm AR is a necessary process for successful sperm-egg fusion. We found that the percentage of AR induced by A23187 in the HFD group is significantly lower than that in the CD group. This finding means that the spermatozoa from mice fed our HFD have a lower fusion frequency with oocytes. This expectation is also supported by our finding of a significant lower pregnancy rate in mated females. Taken together, HFD induced obesity does in fact decrease male fertility.

In an attempt to further unveil how a HFD reduces male fertility, we examined the morphological structure of mice testes through both H&E staining and TEM examination. Testicular morphological analyses suggested that Sertoli cells in the seminiferous epithelium of HFD mice underwent atrophy, and cell adhesion between spermatogenic cells and Sertoli cells became loosely arranged. In agreement with these changes, both TEM testicular analyses of mice fed a HFD and BTB integrity assay showed a BTB disruption.

BTB is a structural barrier between the testicular fenestrated capillaries and the interior of the seminiferous tubules, which is of importance in protecting both the spermatogenic cells against blood-borne noxious agents and the seminiferous epithelium from an autoimmune reaction. On the other hand, the BTB is constituted by the tight junctions between adjoining Sertoli cells. Moreover, occludin, ZO-1, clathrin as well as androgen receptor are used as biomarkers of this entity and are essential contributors to its integrity.

As a member of the nuclear receptor superfamily, the androgen receptor mediates androgen activities [34]. Interestingly, Sertoli cell-specific deletion of the androgen receptor displays a defective BTB, which is coupled with reduced occludin and ZO-1 expression [35, 36]. Furthermore, previous studies showed that males with an *Ar* gene mutation might be at a high risk for infertility [37]. Our results in vivo also demonstrated that the level of androgen receptor expression was reduced and accompanied by declines in testosterone, occludin and ZO-1 levels in mice fed a HFD, which indicates that androgen receptor is likely an upstream factor affecting BTB integrity under the high-fat condition.

**Fig 7:**
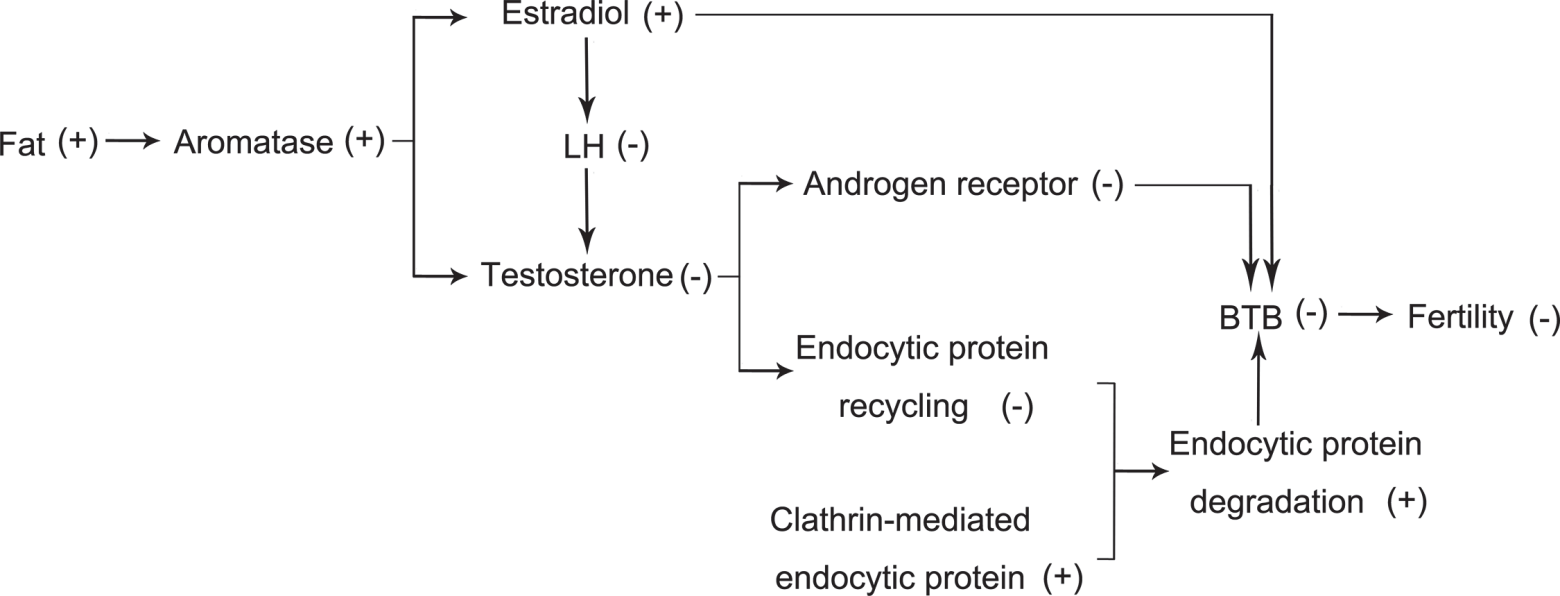
Model accounting for declines in male fertility in mice fed HFD. However, in mice on HFD, what is the impact of BTB integrity disruption on spermatogensis or subsequent sperm maturation? We found that these mice generally have decreased sperm motility and progressive motility, increased rate of teratozoospermia and even lowering of the sperm fertility index. Undoubtedly, the disruption of BTB integrity is one of the crucial factors for this pathogenesis. A possible reason accounting for why mice on a HFD have a disrupted BTB, but without any sperm concentration alteration is that preleptotene/leptotene spermatocytes merely traverse the BTB in advance of BTB integrity disruption rather than later at spermatogenic arrest prior to meiosis onset. Therefore, spermatozoa of HFD mice within the cauda epididymides may be immature eventually, and sperm abnormality rates will also dramatically increase more in the HFD group than in the CD group [16, 18].

The migration of preleptotene/leptotene spermatocytes across the BTB is an important cellular event that occurs during stage Ⅷ of the seminiferous epithelial cycle of spermatogenesis [38]. This event involves intermittent phases of junction disassembly and reassembly between neighboring Sertoli cells to facilitate their transit [39]. On the other hand, the maintenance of BTB integrity at stage Ⅷ of the seminiferous epithelial cycle especially depends on the balance between endocytic vesicle-mediated tight junction proteins (e.g., occludin, ZO-1) transcytosis, recycling and degradation [40, 41]. Previous studies validated that testosterone can regulate BTB integrity through its effects on clathrin-mediated tight junction proteins endocytosis, transcytosis and recycling [42, 43]. Besides,estradiol also has instead negative effects on BTB integrity [40, 44]. Thus, as HFD mice have lower testosterone levels whereas clathrin levels rose, the balance between the tight junction proteins endocytosis, transcytosis, recycling and degradation broke, and much more endosome-mediated tight proteins appeared to be degraded. Therefore, it can be deduced that increased endosome-mediated tight junction proteins degradation is another pathway by which the BTB integrity is disrupted (a diagram shown in Fig 7).

In summary, we show here that diet-induced obesity in male mice can induce a significant impairment of sperm function parameters, including decreased motility, progressive motility, AR percentage, fertility rate and rises in teratozoospermia rate. Moreover, our results also reveal that endocrinopathy disorders in mice fed a HFD suppress male fertility via disrupting BTB integrity. The downstream targets of dysregulated testosterone levels, which disrupt BTB formation and its integrity, are likely to be androgen receptor and clathrin-mediated tight junction proteins, endocytosis, transcytosis and recycling. Thus, elucidation of the mechanism underlying how a HFD impairs male fertility may provide novel therapeutic options for better management of male subfertility in specialized clinics serving diet-induced obese men.

## Supporting Information

S1 Dataset Food intake by mice in CD and HFD groups (g/mouse/week). (XLSX)

S2 Dataset Fertility rate of female mice mated with HFD male mice and CD male mice. (XLSX)

S1 Fig Effects of HFD on mouse testicular morphology. Hematoxylin and eosin-stained testicular sections from mice fed HFD. Scale bars = 50 μm.
(TIF)

S2 Fig Transmission electron micrograph of seminiferous epithelium of mice fed HFD. (A) TEM of seminiferous epithelium of mice fed HFD. Scale bars = 2 μm. (B) Magnified view of indicated section in (A). Scale bars = 1 μm. Sc, Sertoli cell; Spg, spermatogonium; BTB, blood-testis barrier; Straight line show BTB between two adjacent Sertoli cells.
(TIF)

S3 Fig Localization of ZO-1 in seminiferous tubule of mice fed CD and HFD. The arrows indicate ZO-1 protein and dash lines show the basement membrane of seminiferous tubule of mice fed CD and HFD. Scale bars = 20 μm.
(TIF)
