# Supplementary figures and images for: Diet-Induced Obesity in Male C57BL/6 Mice Decreases Fertility as a Consequence of Disrupted Blood-Testis Barrier
Source: PLoS One. 2015 Apr 17;10(4):e0120775. doi: 10.1371/journal.pone.0120775 (PMC4401673; doi:10.1371/journal.pone.0120775)

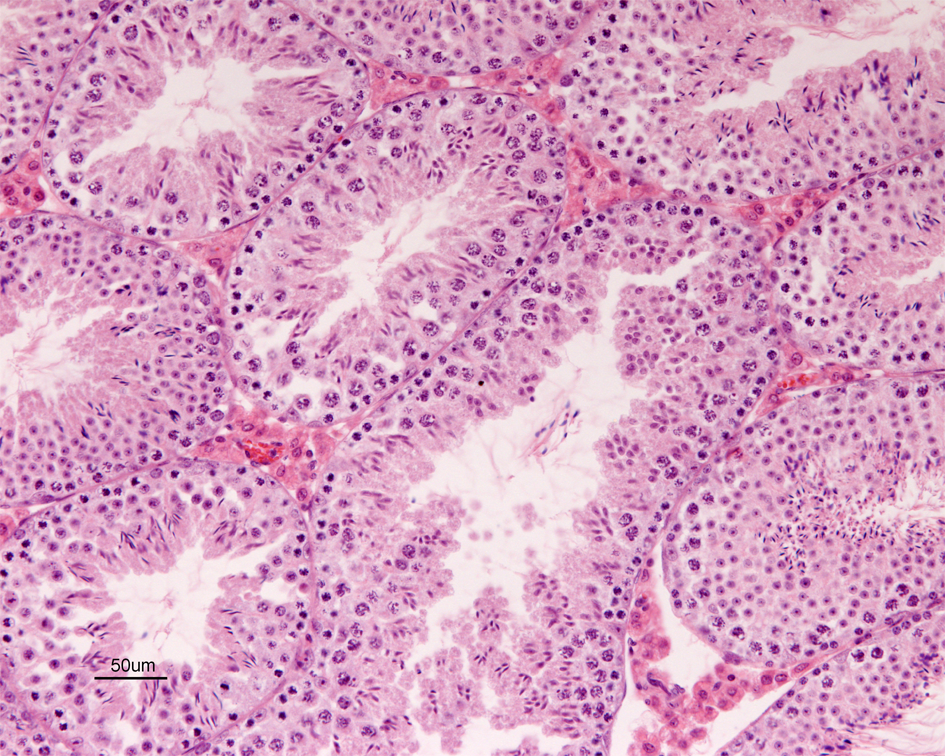

Supplement: S1 Fig — Hematoxylin and eosin-stained testicular sections from mice fed HFD. Scale bars = 50 μm. (TIF) [file pone.0120775.s003.tif]

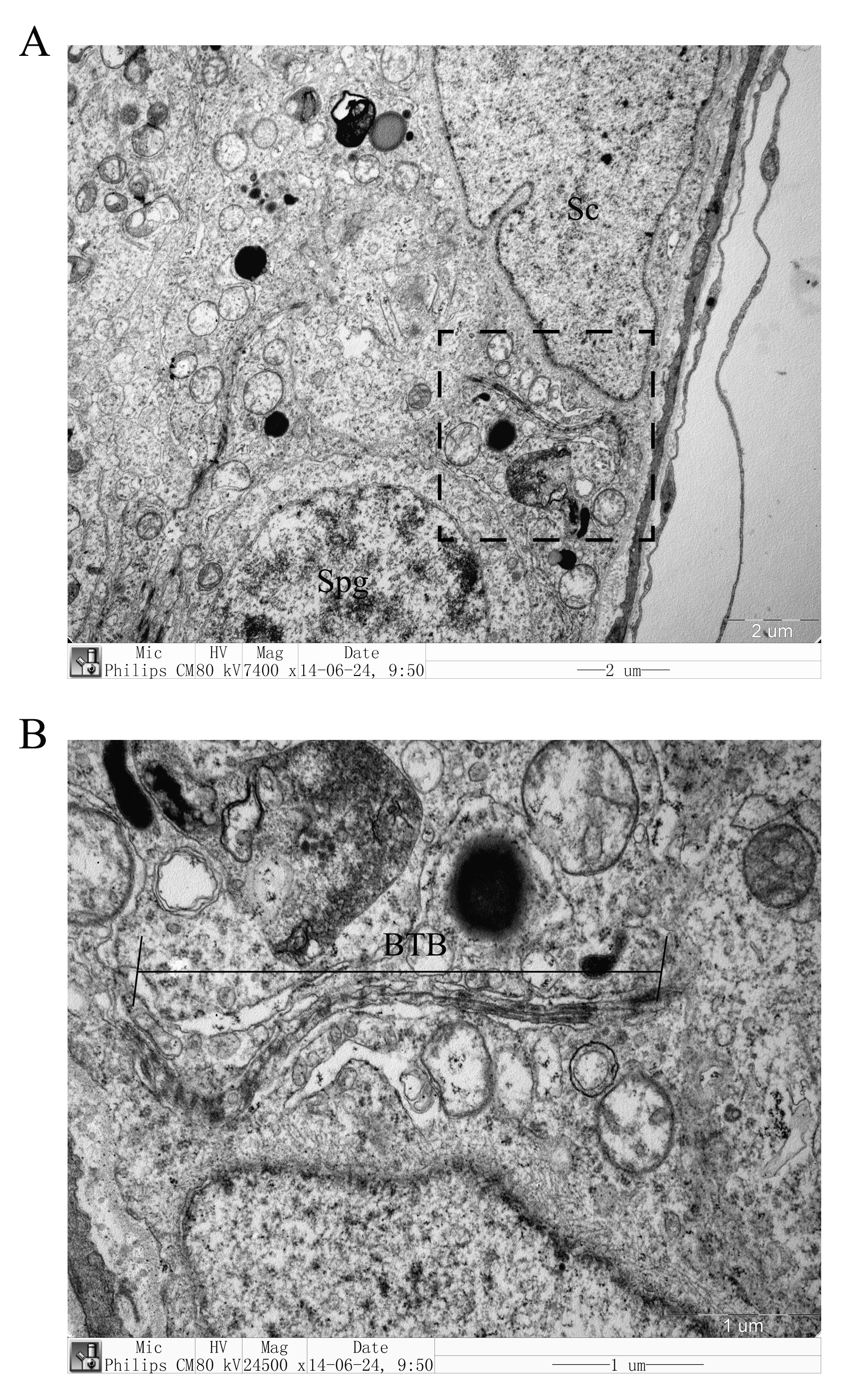

Supplement: S2 Fig — (A) TEM of seminiferous epithelium of mice fed HFD. Scale bars = 2 μm. (B) Magnified view of indicated section in (A). Scale bars = 1 μm. Sc, Sertoli cell; Spg, spermatogonium; BTB, blood-testis barrier; Straight line show BTB between two adjacent Sertoli cells. (TIF) [file pone.0120775.s004.tif]

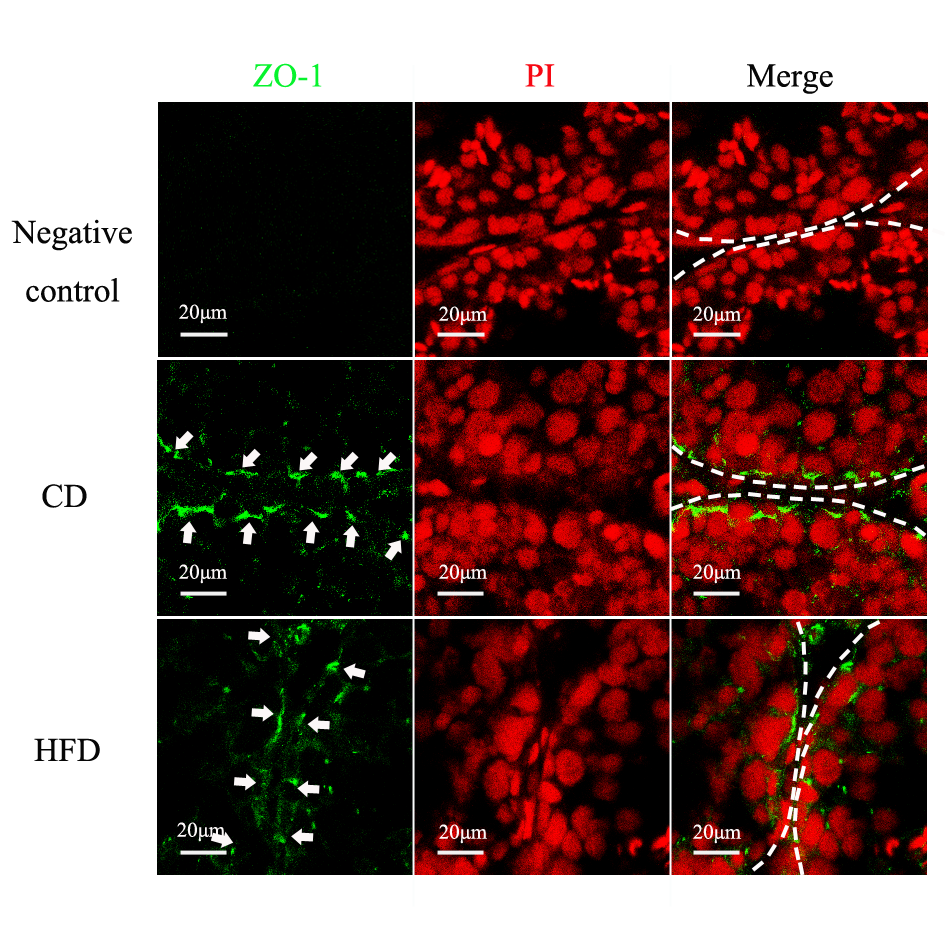

Supplement: S3 Fig — The arrows indicate ZO-1 protein and dash lines show the basement membrane of seminiferous tubule of mice fed CD and HFD. Scale bars = 20 μm. (TIF) [file pone.0120775.s005.tif]
